# Automatic detection of peripheral stimuli in shooters and handball players: an event-related potential study

**DOI:** 10.1007/s00221-021-06071-2

**Published:** 2021-03-09

**Authors:** Bela Petro, Ágota Lénárt, Zsófia Anna Gaál, Petia Kojouharova, Tibor Kökény, Csaba Ökrös, István Czigler

**Affiliations:** 1 Doctoral School of Psychology, Eötvös Loránd University, Budapest, Hungary; 2 Institute of Psychology, ELTE Eötvös Loránd University, Budapest, Hungary; 3 Institute of Cognitive Neuroscience and Psychology, Research Centre for Natural Sciences, Budapest, Hungary; 4 University of Physical Education, Budapest, Hungary

**Keywords:** Handball, Shooting, Peripheral visual sensitivity, Event-related brain potentials (ERPs), Automatic change detection, Visual mismatch negativity

## Abstract

This study examined the practice-related sensitivity of automatic change detection. The visual mismatch negativity (vMMN) component of event-related potentials was compared in handball players and in sport shooters. Whereas effective performance in handball requires processing of a wide visual field, effective performance in shooting requires concentration to a narrow field. Thus, we hypothesized larger sensitivity to peripheral stimuli violating the regularity of sequential stimulation in handball players. Participants performed a tracking task, while task-irrelevant checkerboard patterns (a frequent and an infrequent type) were presented in the lateral parts of the visual field. We analyzed the vMMN, a signature of automatic detection of violating sequential regularity, and sensory components (P1, N1, and P2). We obtained larger vMMN in the handball players’ group indicating larger sensitivity to peripheral stimuli. These results suggest the plasticity of the automatic visual processing, i.e., it can adapt to sport-specific demands, and this can be captured even in a short experimental session in the laboratory.

## Introduction

Stimuli violating the regularity of sequential stimulation are automatically registered, even if such stimuli are different from the task-relevant events. The automatic registration of deviant stimuli is indicated by the mismatch responses of event-related potentials (ERPs) in the auditory (for reviews, see Garrido et al. 2009; Näätänen et al. 2011), in the visual (for reviews, see Czigler 2007; Kimura et al. 2011; Stefanics et al. 2014) and in the somatosensory modality (e.g., Shinozaki et al. 1998; Shen et al. 2018). In the visual modality the mismatch response, the visual mismatch negativity (vMMN) is elicited by simple, deviant visual features (e.g., orientation, spatial frequency, and color), perceptual categories (e.g., symmetry, numerosity, and object-related regularities), higher-order visual (e.g., facial emotion, gender, left vs. right hand), sequential, and even semantic characteristics. VMMN is generated in visual brain areas (within the occipital, temporal or parietal cortices), and according to some research anterior structures are also involved (e.g., Kimura et al. 2010).

One of the aims of the present study was to investigate whether expert knowledge could modify the effect of this kind of automatic change detection. To this end, we compared the sensitivity of vMMN between two groups of experienced athletes: handball players and sport shooters. Successful handball players (as well as athletes in other team sports, like football, hockey, basketball, etc.) have to process simultaneously events in various spatial locations. However, in other sports such as shooting and archery, the nature of relevant information in this respect is just the opposite, and athletes in these sports have to exclude peripheral stimuli. We hypothesized that due to this difference, task-irrelevant events in the visual periphery would elicit larger vMMN in handball players than in shooters, showing a presumed plasticity of the system underlying the automatic detection of potentially irrelevant events.

At the level of attentional processing, experienced athletes in open skill sports (including team sports like soccer, volleyball, and hockey) usually outperform novices in spatial attention tasks (Memmert 2009; Scharfen and Memmert 2019). In team sports such as handball and football, practice is associated with larger attentional sensitivity in the horizontal dimension (Hüttermann et al. 2014). We hypothesized that team sports demand not only high attentional performance, but also high efficiency of automatic visual information processing.

So far, investigations of practice in shooters concentrated on task-related (attentional) processing. Czigler et al. (1998) obtained shorter reaction times in a target-identification task, and larger amplitudes of attention-related ERP components (selection negativity and positivity, and N2b) in elite shooters. Di Russo et al. (2003) reported shorter saccadic latency, and more stable fixation in the presence of distractors in elite shooters. Kim et al. (2019) compared the scanning strategy of archers of the Korean national team with collegiate archers in a procedure with emulating the actual archery situation. In the earlier phase of shooting fixation, duration of the elite archers was longer, and the number of saccades was reduced in comparison to the less experienced archers. This result was similar to the expert-novice difference in other sports (e.g., Castiello and Umiltà 1992; Williams and Davids 1998; Bosel 1998).

Like the majority of vMMN studies, we applied the passive oddball paradigm. In this paradigm, task-irrelevant frequent (standard) and infrequent (deviant) events are presented, while participants perform an attention-demanding task. As the result of such studies, infrequent (deviant) stimuli usually elicit larger posterior negativity than the frequent, standard stimuli. The activity difference is due to two processes. First, stimulus repetition, a kind of sequential regularity, generates adaptation to the specific stimulus features. Second, the representation of the deviant stimuli does not match the representation of the standard stimuli, and this mismatch process manifests itself in a posterior negative ERP component, the visual mismatch negativity. Brain-electric activity related to stimulus-specific adaptation (or repetition suppression) is considered as the automatic buildup of memory processes and the function of this process is to predict the income of possible events (Stefanics et al. 2014). In the framework of predictive coding theory (e.g., Garrido et al. 2009), the additional, deviant-related activity is considered as an error signal. It is important to emphasize that both processes, the stimulus-specific adaptation and the additional deviant-related activity are automatic processes, without the contribution of attention capture or voluntary orientation of attention.

To the best of our knowledge, the only vMMN study comparing participants in automatic visual processing as a function of the amount of physical activity was conducted by Pesonen et al. (2019). In this study, sequences of standard and deviant task-irrelevant stimuli (oblique bars with different orientation) were presented as stimuli, while the participants attended to an audio play. At the posterior locations, the latency of the later part of the deviant minus standard difference potential (207–248 ms in the vMMN component) was earlier in the physically active group (in comparison to an inactive group), but no significant amplitude difference appeared. The authors did not report data on the earlier part of the difference wave that seems to be larger in the active group on their Fig. 2. The instruction to attend to the audio play is unfortunately not particularly useful for distracting attention from visual stimuli (Czigler 2007), therefore, it is possible that the group-related latency difference was due to attentional factors instead of processes of automatic detections.

The second aim of the present study was to develop a paradigm capable of investigating vMMN within a short session. Hence, we applied only 11 electrodes for the EEG recordings. If reliable vMMN emerges in a session within some minutes, this method will be appropriate even in field studies or with participants of limited availability. In the present study, we applied a variety of the method developed by Sulykos et al. (2018). As a substantial difference, in the Sulykos et al. (2018) study, the ERP-related stimuli appeared at the lower part of the visual field, whereas in the present study, these stimuli were presented to the left and right sides of the visual field. This is because processing of stimuli in the horizontal dimension is especially important in some ball sports (Hüttermann et al. 2014), including handball.

In short, we hypothesized that the laterally presented stimuli would elicit larger effects in the handball players’ group than in the shooters’ group, due to the demands for a wider field of information intake. We also investigated whether the P1, N1, and P2 exogenous components (indicating early perceptual activity) were different in the two groups.

## Methods

### Participants

Twenty sport shooters (10 female, mean age: 23.6 years, SD: 6.2) and 20 handball players (11 female, mean age: 20 years, SD: 1.7) with normal or corrected-to-normal vision participated in the study. All participants in the handball players’ group played at national level. Eighteen of the shooters competed at the first class or master level, 2 of them at the second class (national qualification). All athletes practiced at least two times a week. We applied an a priori power analysis to determine the total sample size, with an alpha of 0.05, a power of 0.8, and an effect size of *f* = 0.33. The effect size estimate is based on Sulykos et al. (2018). This resulted in a minimum of sample size of *N* = 19/group. Written informed consent was obtained from all participants prior to the experimental procedure. The study was conducted in accordance with the Declaration of Helsinki and approved by the United Ethical Review Committee for Research in Psychology (EPKEB).

### Stimuli and procedure

**Fig. 1:**
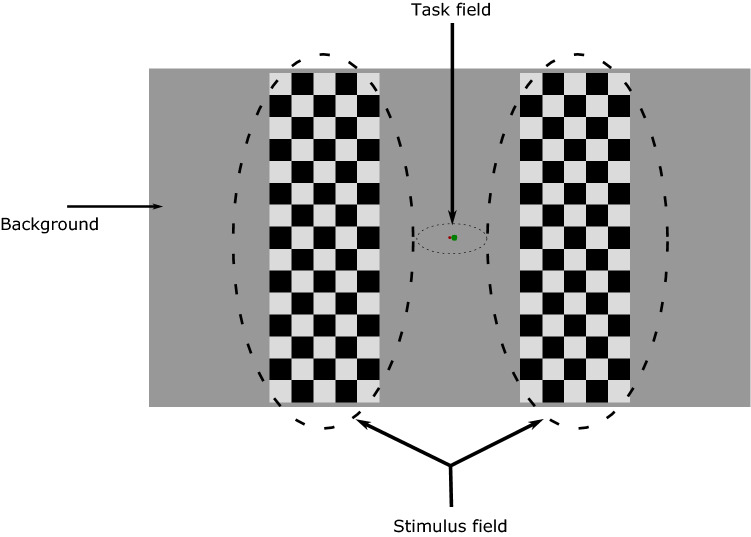
An example of the stimulus display

The stimuli were presented on a 24-in. LCD monitor (Asus VS229na) with a 60 Hz refresh rate. Figure 1 shows the stimulus display. The vMMN-related stimuli were checkerboard patterns presented on the left and right side of the display with a distance of 2.57 degrees of visual arc between them from a 135-cm viewing distance. The size of the patterns was 6 degrees of visual arc vertically and 2 degrees of visual arc horizontally. The pattern consisted of 15 × 5 squares of equal size. The luminance of the bright and dark squares was 81.4 and 0.1 cd/m^2^, respectively. The patterns appeared on a light-grey background of 36.7 cd/m^2^. We created the deviant pattern from the standard by reversing the locations of the dark and bright squares. Both stimulus duration and inter-stimulus intervals were 530 ms ± 50 ms. Within one block, there were 95 stimuli: 76 standards and 19 deviants. Thus, one block was approximately 100 s long. The number of standard stimuli between two deviants was randomly varied between 2 and 8. We also used reversed control blocks where the standard and deviant patterns were interchanged. The participants completed 4 blocks, half of them in ABBA and the other half in BAAB order, where A and B are the ordinary and reversed control blocks.

Participants performed a similar tracking task as in the study of Sulykos et al. (2018). In the middle of the screen between the stimulus fields, there was a red fixation point (diameter: 3 min of visual arc). A green disc (diameter: 6 min of visual arc) moved horizontally across the red fixation point, and the task was to keep the disc on the fixation point using the left and right arrow keys of a computer keyboard. Maintaining the disc on the fixation point, or no more than 0.4 min of visual arc outside the fixation point was considered as being on the fixation point. At a longer distance, the color of the disc changed to blue indicating an error. The task demanded continuous visual attention to the center of the screen as the direction of the moving disc changed randomly.

### Measurement of electrical brain activity

Electrical brain activity was recorded from 11 locations (FZ, P7, P8, PO3, PO4, PO7, PO8, POZ, O1, O2, and OZ) selected from the extended 10–20 system (BrainVision Recorder 1.21.0303, ActiChamp amplifier, Ag/AgCl active electrodes, EasyCap (Brain Products GmbH), sampling rate: 1000 Hz, DC-70 Hz online filtering). The reference electrode was placed on the nose tip, and the ground electrode on the forehead (AFz). Horizontal and vertical electrooculograms (HEOG and VEOG) were recorded with bipolar configurations between two electrodes (placed lateral to the outer canthi of the two eyes and above and below the left eye, respectively). The EEG signal was bandpass filtered offline with a non-causal Kaiser-windowed Finite Impulse Response filter (low pass filter parameters: 30 Hz of cutoff frequency, beta of 12.265, a transition bandwidth of 10 Hz; high pass filter parameters: 0.1 Hz of cutoff frequency). Stimulus onset was measured by a photodiode, providing exact zero value for averaging. Epochs ranging from 100 to 500 ms relative to the onset of stimuli were extracted for further analysis, separately for standards and deviants. The 100-ms pre-stimulus interval of each epoch served as the baseline. Epochs with larger than 100 μV voltage change at any electrode were considered artifacts and rejected from further processing. The individual amplitude values of the ERP components and difference potentials (deviant minus standard) were calculated as the average of the ± 10 ms range around the largest positivity/negativity within their appropriate ranges of the grand average ERPs and difference potentials specified in the “Results”. For illustrative purposes at tables and figures, we created the parietal-occipital (PO: PO3, POZ, PO4) and the occipital (O: O1, OZ, O2) ROIs.

### Statistical analysis

We calculated repeated measures analyses of variance (ANOVA) for the amplitude of the difference potential (vMMN) with the independent factor of Group and within-subject factors of Anteriority (Parieto-occipital, Occipital), and Laterality (Left, Middle, Right). Amplitude and latency of the exogenous components (P1, N1, and P2) were analyzed in similar ANOVAs, with the addition of Stimulus factor (Deviant, Standard). We employed Greenhouse–Geisser correction if the sphericity assumption was violated. For post hoc comparisons, we applied the Tukey HSD test. Effect size was calculated as *η*_*p*_^2^. We applied unpaired Student’s *t* test in the comparison of task performance between the two groups. In this analysis, we applied Cohen’s *d*. We used the Statistica package (Version 13.4.0.14, TIBCO Software Inc.) for statistical analysis.

## Results

### Behavioral results

Performance in the tracking task was expressed as the number of color changes of the disc, i.e., when the disc was outside of the target field. The mean of such erroneous events in the four blocks was 13.6 (*S.E.M.* = 2.6) and 18.15 (*S.E.M.* = 3.8) in the shooters’ and handball players’ group, respectively. According to the independent-sample *t* test, the difference was not significant, *t*(38) = 0.99, *p* = 0.33, Cohen’s *d* = 0.31.

### Event-related potentials

After artifact rejection, the number of averaged ERPs for deviants was 69.3 (S.E.M. = 1.4) for shooters, and 71.0 (S.E.M. = 1.2) for handball players, respectively. The similar data for standard stimuli were 279.7 (S.E.M. = 4.9) and 284.1 (S.E.M. = 4.5) for the shooters and handball players, respectively. Accordingly, there was no difference between the groups in the numbers of averaged responses (in *t* tests *p* > 0.4 in both cases).

**Fig. 2:**
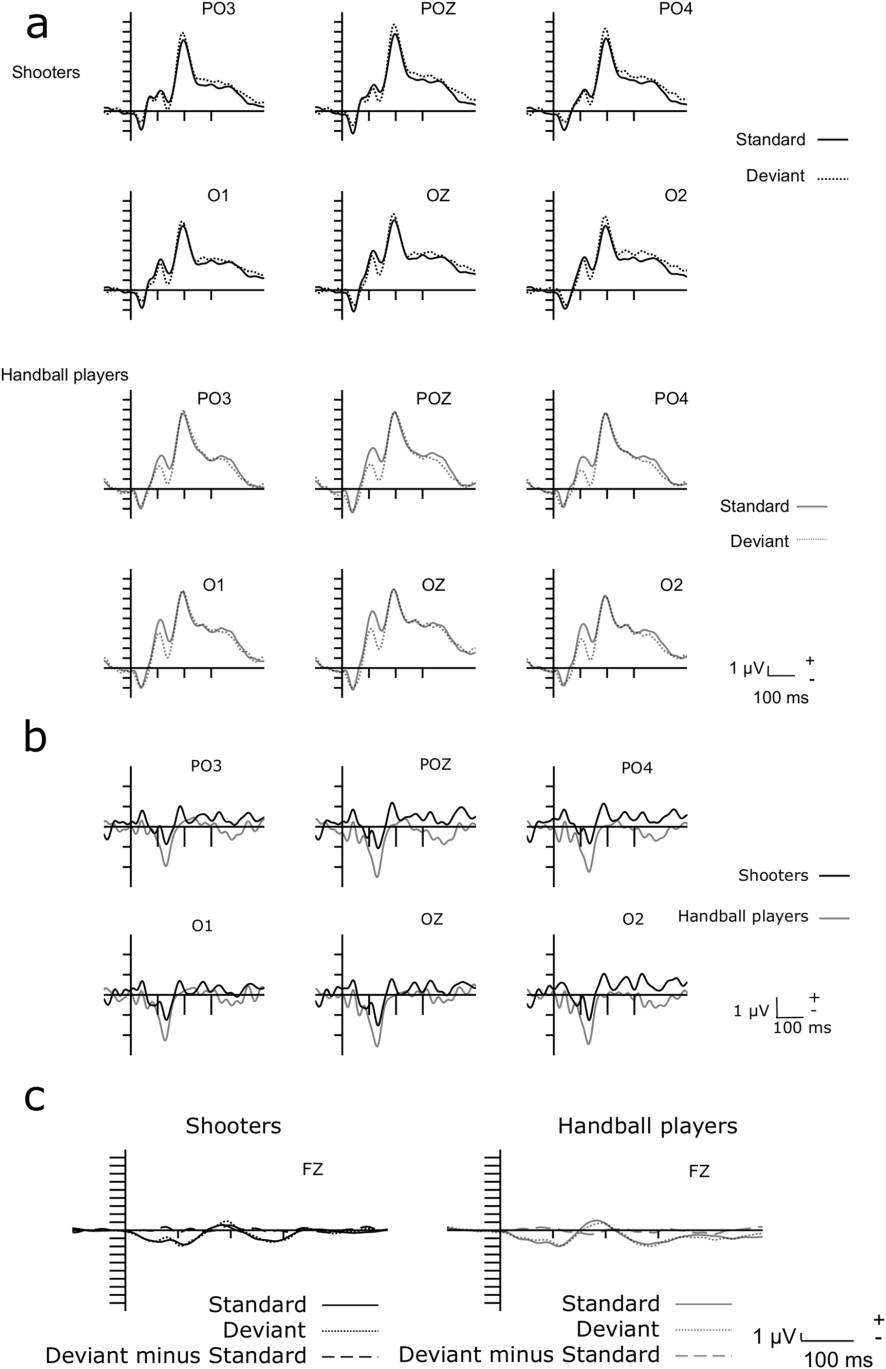
ERPs to the standard and deviant stimuli over 6 posterior electrode locations in the two groups (shooters above, handball players below) (**a**), deviant minus standard difference potentials over the 6 posterior electrode locations (**b**), ERPs to deviant and standard stimuli, and the deviant minus standard difference potential at FZ location (**c**)

Figure 2a shows the ERPs to the standard and deviant stimuli over the posterior locations. According to Fig. 2a, the stimuli elicited an early posterior negative wave, identified as C1 (Di Russo et al. 2002), the P1, N1, and P2 components.

Table 1 shows the P1 and N1 mean amplitude and latency values for the standard and deviant stimuli in the PO (PO3, POZ, PO4) and O (O1, OZ, O2) ROIs in the two groups. The standard errors of the means (S.E.M.) are in parenthesis.

**Table 1:** P1 and N1 mean amplitude and latency values in the PO and O ROIs (S.E.M. in parenthesis)

	Parieto-occipital		Occipital	
	Standard	Deviant	Standard	Deviant
Amplitude				
P1				
Shooters	2.20 (0.59)	1.94 (0.79)	3.33 (0.63)	2.84 (0.76)
Handball players	3.51 (0.76)	2.13 (0.62)	4.88 (0.89)	3.32 (0.79)
N1				
Shooters	1.55 (0.35)	0.80 (0.35)	2.30 (0.40)	1.29 (0.34)
Handball players	2.52 (0.71)	0.76 (0.80)	3.19 (0.85)	1.46 (0.95)
Latency				
P1				
Shooters	109.77 (3.99)	107.78 (3.60)	115.02 (3.72)	114.27 (2.82)
Handball players	115.27 (4.16)	108.27 (4.53)	115.02 (3.24)	107.57 (3.80)
N1				
Shooters	126.50 (4.41)	127.53 (4.21)	126.67 (5.05)	127.47 (4.19)
Handball players	136.42 (5.16)	133.55 (4.19)	138.37 (4.82)	135.18 (4.53)

**Fig. 3:**
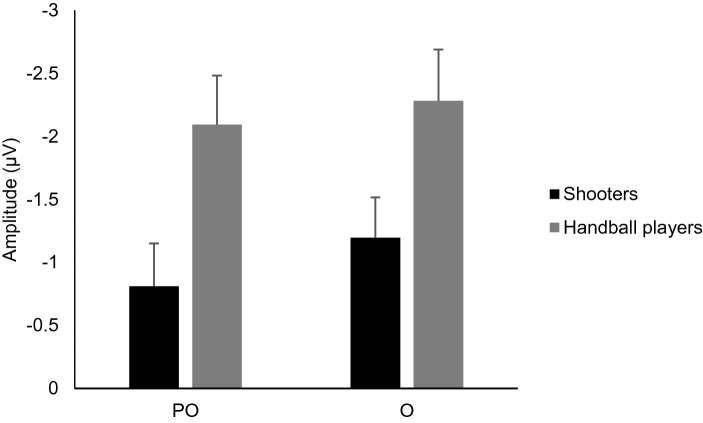
The mean amplitude values of the deviant minus standard difference potentials in the two groups in the PO and O ROIs (the error bars show S.E.M.)

Figure 2b shows the deviant minus standard difference potentials over the posterior locations. In the 100–150 ms range, the difference potential was negative, and larger in the handball group. Note, that a small positive-going deflection is an indication of a process within the P1 and the N1 range. Deviant minus standard difference potentials (the vMMN component) were calculated over the PO3, POZ, PO4, O1, OZ, and O2 locations. In an ANOVA with factors of Group (shooters, handball players), Anteriority (PO, O) and Laterality (left, midline, right), we obtained a main effect of Group, *F*(1,38) = 5.41, *η*_*p*_^2^ = 0.12, *p* = 0.025, a main effect of Anteriority, *F*(1,38) = 9.26, *η*_*p*_^2^ = 0.19, *p* = 0.004, and a main effect of Laterality, *F*(2,76) = 4.57, *η*_*p*_^2^ = 0.11, *ɛ* = 0.72, *p* = 0.024. According to the Tukey HSD test, the latter effect was due to the smaller difference potential amplitude on the left side than in the midline. The amplitude differences of the deviant minus standard difference potentials between the two groups in the parietal-occipital (PO: PO3, POZ, PO4) and occipital (O: O1, OZ, O2) ROIs are presented in Fig. 3.

In ANOVA results on the amplitude and latency values of the exogenous components, we report only effects relevant for group and stimulus differences. P1 amplitude was measured as the largest positivity within the 80–140 ms range of the grand average ERPs. In an ANOVA with factors of Group (shooters, handball players) × Stimulus (deviant, standard) × Anteriority (PO, O) × Laterality (left, midline, right), we obtained a main effect of Stimulus, *F*(1,38) = 13.03, *η*_*p*_^2^ = 0.26, *p* < 0.001. The Stimulus × Group interaction was also significant, *F*(1,38) = 4.61, *η*_*p*_^2^ = 0.11, *p* = 0.038. According to the Tukey HSD test, the standard stimuli elicited larger P1 than the deviants in the handball players’ group. In a similar ANOVA on P1 latency, we obtained a significant main effect of Stimulus, *F*(1,38) = 8.85, *η*_*p*_^2^ = 0.19, *p* = 0.005. This effect was qualified by the Stimulus × Group interaction, *F*(1,38) = 4.12, *η*_*p*_^2^ = 0.10, *p* = 0.049. According to the Tukey HSD test, in the handball players’ group, the deviant-related P1 appeared earlier.

The N1 component emerged superimposed on a long-lasting positivity as a reduced positive value. N1 amplitude was measured as the largest negative/smallest positive value within the 100–200 ms range. In a similar ANOVA, the main effect of Stimulus was significant, *F*(1,38) = 24.19, *η*_*p*_^2^ = 0.39, *p* < 0.001. The N1 amplitude was smaller (less positive) in the deviant condition. Concerning N1 latency, we obtained neither group nor stimulus-related significant effect.

In similar measures on the P2 amplitudes and latencies (150–250 ms range), the Group and Stimulus factors did not contribute to any significant effect. As Fig. 2c shows, there were neither group nor stimulus-related differences over the FZ location.

## Discussion

In this study, we compared the sensitivity of automatic detection of peripheral and task-irrelevant events violating the regularity of sequential stimuli in handball players and shooters. The tracking task required continuous fixation. Since both groups performed on a high level in this task, we assume this control of attention to be appropriate.

As the most important result of the present study, the deviant minus standard difference potential appeared as a posterior negativity in the ~ 100–150 ms range. The deviant minus standard difference potential is an aggregate of two processes: decreased activity to the standard stimulus (stimulus-specific adaptation; SSA), and the increased activity to the processing of deviant stimuli (‘genuine’ vMMN, Kimura et al. 2009). In case of stimulus-specific adaptation, one may expect also the reduction of the exogenous P1. In fact, it was not the case. Therefore, we consider the negative difference potential is mainly a consequence of vMMN emergence.

The predictive coding theory is a widely accepted explanation of the activity underlying auditory and visual mismatch components (e.g., Garrido et al. 2009; Stefanics et al. 2014). According to this account, the representation of environmental regularity (in this case the standard checkerboard) predicts the characteristics of expected events. Simple stimuli like the localization of the elements of checkerboard patterns are represented within the posterior cortical regions (Urakawa et al. 2010; Susac et al. 2014). A mismatch between the representations of incoming and expected stimuli generates an error signal, and the error signal is processed until it matches the updated representation. The ‘genuine vMMN’ (e.g., Kimura et al. 2009) is a signature of such processes.

The present results can be interpreted as the larger attentional sensitivity in the horizontal dimension in the group of handball players (Hüttermann et al. 2014). In case of more efficient overt orientation to the checkerboard pattern, the obvious expectation is the increased number of saccadic eye-movement. Contrary to this possibility, the numbers of averaged epochs of both standard and deviant stimuli were equal in the two groups, i.e., the mostly eye-movement-related artifact-rejection rate did not increase in the handball player group. In addition, in a vMMN study with eye-movement recording, fixation to the field of the tracking task was fairy effective (File and Czigler 2019). In case of both overt and covert attentions, the emergence of attention-related ERP components, like P3a at anterior locations is expected (e.g., Polich 2007). As ERPs at the FZ location show, there were neither group nor stimulus-related differences at this location.

Although the task-irrelevant checkerboard patterns elicited ‘canonical’ exogenous ERP components (P1, N1, and P2 components), in this study, the identification of amplitude and latency values of the P1 and N1 components are not without problem. This is because the latency range of the largest positivity and negativity overlapped. Therefore, the small P1 latency difference (earlier P1 to deviant stimuli in handball players), together with the lack of latency effects on the N1 component cannot be considered as a reliable indicator of earlier onset of perceptual processing in any of the groups. The possibility of earlier P1 latency (i.e., faster responses of early perceptual structures) deserves further studies.

We assume the larger sensitivity to deviant stimuli in handball players is related to the difference between the automatic (pre-attentive) information processing of the two groups, and this difference is a signature of the plasticity of the processing system underlying the investigated aspects of change detection. Alternatively, to put it another way, shooters were less susceptible to the distractor effects of irrelevant stimulation. These results show that the specific demands of various sport activities are present not only in attentional performance, but also in the fields of automatic (pre-attentive) processing. Note, that this group difference is not necessarily the result of the sport-specific trainings, but the differences in automatic change detection might have predated sports training, predisposing athletes to eventual expertise in a sport that matched their advantage in automatic information processing. However, the adherence to a particular sport by adolescents depends on so many factors that automatic information processing is very unlikely to play a decisive role. Thus, we tend to think that the observed superiority of handball players in automatic, peripheral change detection is due to long-term sport-specific training, hence a manifestation of plasticity of the system.

As an important aspect of the present study, a very short session (less than 20 min., including preparation for EEG recording) was enough to obtain group-related differences, and the preparation time can be further reduced using only 6 electrodes reported here. As a next step for using this vMMN method in applied settings would be the investigation of individual differences and the changes of individual recordings as a possible correlate or predictor of athletic performance.

## Data Availability

The datasets generated during and/or analyzed during the current study are available from the corresponding author on reasonable request.

## Code availability

The matlab code for stimuli presentation is available from the corresponding author on reasonable request.
